# Digitally-Compensated Wideband 60 GHz Test-Bed for Power Amplifier Predistortion Experiments

**DOI:** 10.3390/s21041473

**Published:** 2021-02-20

**Authors:** Martin Pospíšil, Roman Maršálek, Tomáš Götthans, Tomáš Urbanec

**Affiliations:** Department of Radio Electronics, Brno University of Technology, 61600 Brno, Czech Republic; xpospi29@stud.feec.vutbr.cz (M.P.); gotthans@feec.vutbr.cz (T.G.); urbanec@feec.vutbr.cz (T.U.)

**Keywords:** millimeter-wave systems, I/Q mismatch compensation, digital predistortion, RF impairments

## Abstract

Millimeter waves will play an important role in communication systems in the near future. On the one hand, the bandwidths available at millimeter-wave frequencies allow for elevated data rates, but on the other hand, the wide bandwidth accentuates the effects of wireless front-end impairments on transmitted waveforms and makes their compensation more difficult. Research into front-end impairment compensation in millimeter-wave frequency bands is currently being carried out, mainly using expensive laboratory setups consisting of universal signal generators, spectral analyzers and high-speed oscilloscopes. This paper presents a detailed description of an in-house built MATLAB-controlled 60 GHz measurement test-bed developed using relatively inexpensive hardware components that are available on the market and equipped with digital compensation for the most critical front-end impairments, including the digital predistortion of the power amplifier. It also demonstrates the potential of digital predistortion linearization on two distinct 60 GHz power amplifiers: one integrated in a direct-conversion transceiver and an external one with 24 dBm output power.

## 1. Introduction

With increasing spectral demands, the use of millimeter-wave (mm-wave) frequencies is envisaged in the near future [1]. The potential of several mm-wave bands has been investigated for mobile or vehicular applications [2,3], with the 28 GHz band becoming a part of the 5G New Radio (NR) standard, while the 60 GHz band is promising in terms of its unlicensed access to spectra [4]. The mm-wave bands beyond 52 GHz are currently under investigation for use in 5G NR Release 17 [5]. Due to the excellent resolution they offer, mm-waves have also shown their potential for precise localization [6]. In contrast to the currently most-used microwave frequencies, usually in the sub-6 GHz band, mm-waves offer channel bandwidths in the range of hundreds of MHz. In total, more than 11 GHz of bandwidth is available in mm-waves if both the licensed as well as the unlicensed parts of the spectrum are included.

The Orthogonal Frequency Division Multiplex (OFDM) is currently the most widespread technique applied in wideband wireless communication standards including LTE, 5G or WiFi local area networks, while for point-to-point links, the single-carrier M-ary Quadrature Amplitude Modulation (MQAM) is often used. In the last few years, several waveforms with reduced out-of-band emissions have been designed as complements or envisaged successors of OFDM, such as the Filter Bank Multi Carrier (FBMC) [7], the Generalized Frequency Division Multiplexing (GFDM) [8,9] or the Filtered OFDM (F-OFDM) [10].

Important research activities dealing with the effects of radio frequency (RF) front-end impairments [11] on various communication waveforms have been carried out recently. The outcomes of theoretical analyses, together with impairment compensation techniques, have been thoroughly experimentally verified at microwave frequencies [12,13,14]. Although some early-bird mm-wave setups have already been used for experiments with multicarrier signals [15] and some completely new software defined radios for mm-waves have been built [16], there are still many works [17] dealing with advanced multicarrier waveforms and RF front-end compensation by computer simulations only, often due to the lack of affordable instrumentation for mm-waves.

One of the most severe RF front-end impairments is power amplifier (PA) nonlinearity [18]. This results not only in out-of-band radiation that is actually less problematic in the mm-waves due to the spatial separation of the links, but also in in-band signal distortion that needs to be compensated. Such in-band distortion manifests in the increased spreading of constellation points, which can be quantified by the Error Vector Magnitude (EVM) metric [19]. A perfect way of compensating for PA nonlinearity is to use the digital predistortion (DPD) method for linearization [20,21]. In order to track the changes of the component parameters and their dependency on the center frequency, output power or working temperature, DPD needs to be implemented in an adaptive manner, imposing the requirements on the additional circuitry (an observation receiver is necessary) and signal processing.

Despite the continuing research into advanced front-end architectures [22], a direct conversion (homodyne) concept is still very attractive due to its reduced cost and easy on-chip integration without bulky external filters. On the other hand, the direct conversion architecture [23] suffers from the modulator/demodulator phase and gain imbalances. The spectral images arising due to these impairments result in a significant reduction of the achievable rate, as has been shown in [24] and as we further developed experimentally for the case of the 60 GHz transceiver [25].

Another important RF front-end impairment is phase noise, especially for commercial RF transceiver designs with a low-cost variable frequency synthesis and wide signal bandwidth. In contrast to the gain and phase mismatch or PA nonlinearity [26], receiver performance degradation due to phase noise can be eliminated only by careful design; this is especially true for high-quality frequency synthesis [27]. To a certain extent, the effect of phase noise can be reduced by increasing the symbol time; e.g., by using an increased number of subcarriers in the OFDM system [28]. Otherwise, only a constant phase rotation due to phase noise can be reduced, but not the random time fluctuation of the received symbols.

Note that the influence of the above-mentioned transceiver imperfections is further accentuated in the wideband channels of the mm-wave systems. To date, for wider bandwidths, analog predistortion has usually been employed [29]. A smart combination of analog tunable delay lines has also been recently used for the compensation of 60 GHz beamformers, as presented in [30]. In recent years, increased attention has been paid to the application of digital predistorters [31], including for wideband signals. That makes DPD attractive for mm-wave engineers. The state-of-the-art experimental research is mainly based on expensive universal measurement devices such as high-speed arbitrary waveform generators (AWG) and real-time spectrum analyzers or fast oscilloscopes [32,33,34]. A multi-Gb/s general-purpose AWG and oscilloscope have also been used in a recent demonstration of DPD in the 28 GHz band [35]. The use of such general-purpose equipment significantly increases the costs of the overall system and prohibits the wider deployment of predistortion in commercial mm-wave applications. Alternatively, some works have evaluated the performance of DPD using simulations or models scaled to lower frequency bands [36]. Some of the existing test-beds, such as our time-domain channel sounder [37], are able to process the wide bandwidths of several GHz, but without the capability of transmitting I/Q data, whereas other mm-wave setups allow for arbitrary I/Q data transmission, but with the bandwidths limited far below 100 MHz [15].

In this paper, we present our wideband (up to 900 MHz bandwidth without predistortion) experimental 60 GHz transmitter test-bed with an observation receiver (denoted below simply as the receiver), which was developed not using expensive laboratory instruments [32,33,34,35] but with affordable development kits and discrete components selected to target low-phase noise and sampling clock jitter. The described test-bed is fully MATLAB-controlled and, in contrast to similar recent setups, it contains digital compensation for the RF front-end including amplitude and phase mismatch and power amplifier linearization using digital predistortion. Thus, as summarized in Table 1, the presented test-bed achieves a much higher transmission power, with a bandwidth and linearity performance comparable to or exceeding the state-of-the art works in [38] or [16]. In addition to our recently published conference contribution [39] or our recent paper showing early predistortion results [40], this manuscript presents a much more detailed description of the hardware as well as the compensation algorithms and also provides in-depth results of the digital predistortion application for three examples of typical communication waveforms—MQAM, OFDM and FBMC—and two cases of power amplifiers.

## 2. Hardware of the Test-Bed

**Figure 1:**
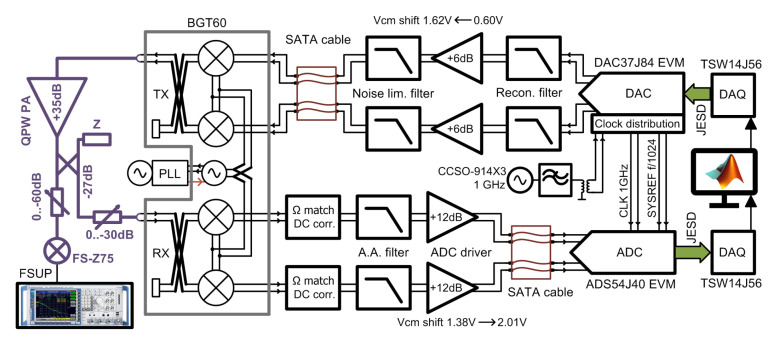
A block diagram of the described mm-wave 60 GHz test-bed. SATA: Serial ATA; ADC: Analog-to-Digital converter; DAC: Digital-to-Analog converter.

**Figure 2:**
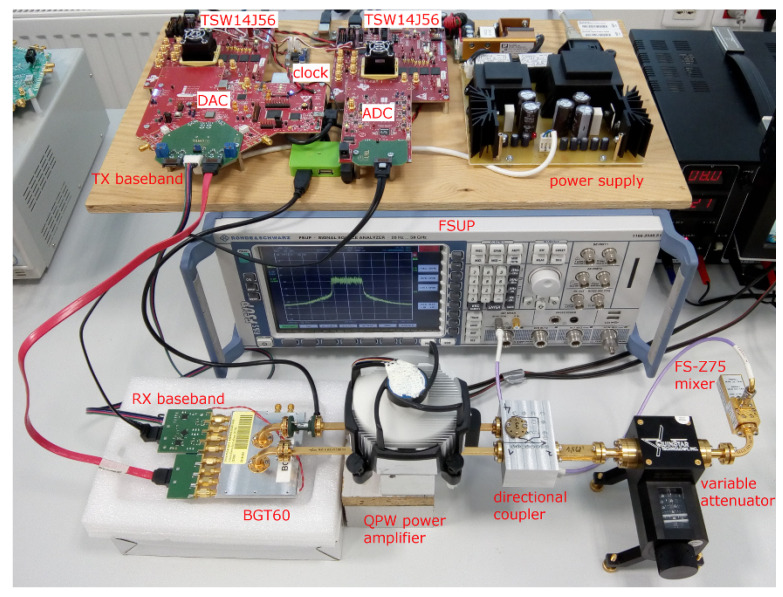
Photo of the described mm-wave 60 GHz test-bed.

A block diagram and a photo of our digitally compensated test-bed for the 60 GHz band are shown in Figure 1 and Figure 2, respectively. As an evolution of our previous narrowband setup [41], the setup is also based on the Infineon BGT60 evaluation board [42]. In contrast to the CompuGen/CompuScope cards with maximal sampling frequencies of 300/400 MHz which we used in the previous setup, the described test-bed is based on the development kits of commercially available Analog-to-Digital (A/D) and Digital-to-Analog (D/A) converters from Texas Instruments accompanied with FPGA data acquisition cards. We developed application interface libraries for MATLAB to fully control BGT60 and data converter parameters from a host PC. The baseband data processing (signal generation, demodulation) and control of the test-bed thus proceeds completely through the MATLAB environment. Each of the crucial parts of the test-bed is described in the following sections.

The whole test-bed uses DC-coupled fully differential signaling, as it offers many benefits over a single-ended design; namely, ground returns and noisy grounds are less of an issue. Moreover, DC offset compensation is sufficiently stable for a standard range of lab temperatures and does not require re-calibration before each measurement. Due to mechanical reasons (the waveguide system at RF ports), baseband units with A/D and D/A converters are connected to the RF front-end (BGT60) via the baseband cables. Despite the unstable contact resistance being sensitive to movements of the cable connections, the lack of available differential 100 Ω cables with connectors led us to use standard Serial ATA (SATA) cables for this application.

### 2.1. RF Section

The whole RF section (depicted in violet on the block diagram in Figure 1) was built from standard WR-15 waveguide components. Waveguides were preferred over coaxial cables due to their better phase stability. To allow for the estimation of RF impairments, an observation receiver is necessary. For this reason, part of the signal from the transmitter (TX) output is coupled back to the BGT60 receiver (RX) port by a directional coupler. The directional coupler had to be designed and manufactured in-house due to the BGT60’s non standard TX/RX port spacing of 0.96 inches. Its design was described in detail in our previous work [43]. The BGT60 output signal is also monitored with the Rohde and Schwarz spectrum analyzer FSUP equipped with an FS-Z75 harmonic mixer.

The experiments with digital predistortion were carried out on two power amplifiers—an internal power amplifier integrated within the Infineon BGT60 transceiver module and an external QuinStar power amplifier QPW QPW-50662330. More details about the power amplifiers can be found in Section 4.1, together with digital linearization performance results. Note that the attenuators are used at the input/output of PA and at the input of the observation receiver to adjust the power levels.

### 2.2. BGT60 Transceiver

The BGT60 chip [42] is a V-band direct conversion transceiver with more than 1 GHz bandwidth, manufactured by Infineon in SiGe technology. It features a fully differential design, an integrated Voltage Controlled Oscillator (VCO) with a prescaler-divider and a 12 dBm (1 dB compression) integrated PA. The BGT60 can be operated in full-duplex mode, but only on the same RF frequency (sharing the integrated VCO). This perfectly suits the intended application, where the receiver serves as the observation path to estimate transmitter impairments. The VCO supports an RF frequency range of 57–64 GHz, controlled by the analog tuning voltage. The Phase Locked Loop (PLL) is external to the transceiver chip, and the one populated on the BGT60 evaluation board is based on the ADF4158 circuit. A 40 MHz PLL reference clock is taken from an on-board Temperature Compensated Crystal Oscillator (TCXO) and is thus non-coherent to the rest of the system. The frequency of the Phase Frequency Detector (PFD) is 20 MHz, and a loop filter is designed for a 2.5 mA current. The baseband ports of TX have 100 Ω input impedance; however, the RX baseband ports need to be loaded with 400 Ω impedance, which complicated the process of matching to standard 100 Ω filters and cables.

In the framework of our cooperation with the Institute of Telecommunications, TU Wien, Austria, we measured the phase noise performance of the BGT60 transmitter using a Rohde & Schwarz signal and spectrum analyzer FSW with a phase noise measurement option. The measured results are shown in Table 2, together with the values specified by the BGT60 datasheet. The data are in good agreement.

### 2.3. Analog to Digital and Digital to Analog Converters

To profit from BGT60’s baseband bandwidth of approximately 1 GHz, we chose a sampling rate of 1 Gsps (complex baseband) for both the D/A converter (DAC) and A/D converter (ADC). We selected the ADS54J40 (dual channel, 14 bit resolution, maximal sampling rate of 1.0 Gsps) and the DAC37J84 (quad channel, 16 bit resolution, maximal sampling rate of 1.6 Gsps) as the ADC and DAC, respectively.

To facilitate implementation, we used the evaluation modules from Texas Instruments for both the ADS54J40 and DAC37J84 converters with some necessary changes. The default analog physical interface of these boards—the passive baluns and SMA connectors—were removed from the modules, and custom analog interface boards were designed to provide differential connections to the DAC and ADC. The custom analog interface boards have physical dimensions enabling them to fit over the analog area of the original D/A and A/D converter boards. In the case of the DAC, this custom analog interface board also contains the TX baseband circuitry described in Section 2.4, while the ADC only serves to connect ADC inputs to a SATA cable and the RX baseband circuitry is joined to the BGT60 module, as shown in Figure 2.

In the digital domain, each DAC and ADC module is connected to an Altera Arria V FPGA-based TSW14J56 pattern generator/data capture card from Texas Instruments, populated with DDR3 SDRAM memory. For a 16 bit sample width, the capacity of 32 Gb of SDRAM allows us to store up to 2 Giga-samples for both transmitted and received signals. The pattern generator cards are connected to the host PC via a USB 3.0 interface.

### 2.4. Baseband Signal Circuity

The signal at the outputs of the D/A converters is passed through an in-house made board with a pair of reconstruction filters, amplifiers and noise-limiting filters. This interface board is physically located at the DAC printed circuit board (PCB) and connected to the BGT60 evaluation board with a SATA cable. The reconstruction filter is a fifth-order passive elliptic filter. The DAC is loaded with a load impedance $R_{L}$ = 25 Ω single-ended (50 Ω differential) and matched to the filter’s load $R_{L}$ = 100 Ω by a resistor divider. The common-mode voltage at the reconstruction filter is 0.60 V. The gain stage follows, based on an LMH5401 circuit, providing a necessary common-mode shift to 1.62 V for BGT60’s TX baseband (BB) ports. This also improves reconstruction filter matching and serves to isolate the filter from the BGT60. The amplifier is configured for a minimum stable gain of 6 dB (including termination loss), as gain boost is not needed in the TX baseband path. The usable bandwidth is well over 4 GHz in this configuration, which is more than sufficient with respect to BGT60 bandwidth. A simple third-order elliptic noise-limiting filter is used after the gain stage to limit wideband noise and to improve the overall lowpass frequency response. The TX baseband path as a whole has a cutoff frequency of approximately 480 MHz (at −6 dB) with about 20 dB gain drop at a Nyquist frequency of 500 MHz.

Conversely, the downconverted baseband signal from the BGT60 receiver is first filtered by a pair of discrete anti aliasing (AA) filters, amplified by a 12 dB gain stage and then passed to the ADC board through a SATA cable connection. The board with these RX baseband components is mounted directly on the BGT60 module to prevent noise pickup in sensitive high-impedance traces. A seventh-order elliptic filter designed for 100 Ω characteristic impedance, with a cutoff frequency of 450 MHz (−6 dB), is used as the AA filter. Besides gain, the ADC driver and the gain stage based on the LMH5401 circuit also provides a common mode shift from 1.38 V to 2.01 V.

In order to match the 400 Ω impedance of the BGT60 RX baseband outputs to the AA filter input impedance, an active matching stage (400 to 100 Ω) based on the ADA4960 circuit was tested in the initial phase of design. The results were unsatisfactory, with either too high gain with respect to remaining circuitry or problems with stability when the gain was lowered. Therefore, the active matching stage was replaced by a passive resistive impedance matching circuit, compromising the overall baseband RX gain by a value of 6 dB. Although this solution resulted in the inability to fully drive the ADC (−7 dBFS with receiver driven to 1dB compression) and the reduction of the available dynamic range, the receiver chain still provides a 65 dB Spurious Free Dynamic Range (SFDR). Note that the most significant spurious signals are located at ±250 MHz, as the actual sampling frequency of the ADS54J40 interleaving (4 × 250 Msps) A/D converters. A further significant contribution to spurious emissions comes from a JESD interface reference clock to FPGA (250 MHz).

### 2.5. Sampling Clock

High-speed acquisition systems come with strict requirements regarding the jitter of the sampling clock source [44]. It is desirable to have significantly lower sampling clock jitter than aperture jitter of the ADC to avoid the available Signal-to-Noise Ratio (SNR) of the ADC being degraded. A standard solution for generating sampling clocks in the 1–3 GHz range is to use PLL and a Voltage Controlled Temperature Compensated Crystal Oscillator (VCTCXO) in the typical range of 100 MHz as a reference. This was also the default solution for the used ADC and DAC evaluation boards, with a 122.88 MHz reference oscillator and LMK04828 chip as a PLL/clock distributor. A broad range of output frequencies can be generated by two internal VCOs (2 or 3 GHz core) and appropriate dividers.

If variable clock frequency is not needed, lower jitter can be achieved using a dedicated oscillator at a fixed frequency. We adopted this solution, which also avoids the use of PLLs in the clock system, in our test-bed. The LMK04828 then serves as a clock distributor only.

**Figure 3:**
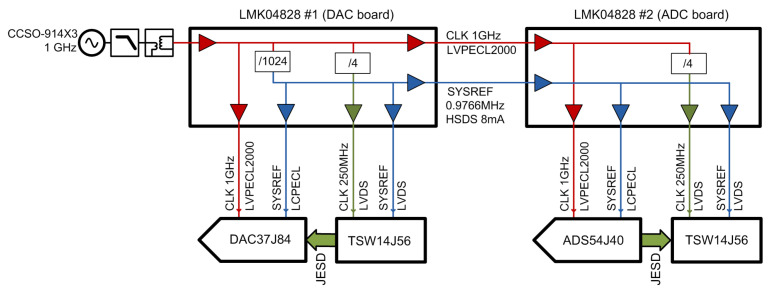
Clock tree.

A clock tree block diagram of the test-bed is shown in Figure 3. The sampling clock source is the ultra-low-phase-noise SAW (Surface Acoustic Wave) oscillator Crystek CCSO-914X3 (1 GHz) with a sinusoidal output. The clock signal is filtered by a simple low-pass filter to suppress higher harmonics (there are no subharmonics). A balun is used to create a differential clock routed to the DAC board. After this point, all clocks down the clock tree are differential. The secondary clocks (a 250 MHz FPGA interface clock and f/1024 periodic SYSREF) are derived from this 1 GHz clock source in the 1st clock distributor LMK04828#1 located on the DAC evaluation board. Through unused clock outputs on the DAC board, the main clock and SYSREF are forwarded to the 2nd clock distributor LMK04828#2 present on the ADC board. Note that several distinct digital voltage standards are used to meet the different common-mode voltage requirements of the individual circuits. The clock to data converters are DC coupled and use LVPECL and LCPECL, while clocks for the FPGA boards use LVDS.

The phase noise and the RMS jitter of the sampling clock for the standard PLL-based and selected fixed frequency solutions with SAW oscillators are summarized in Table 3. According to our simulations, the achievable RMS jitter of the manufacturer’s default solution with PLL would approximately be 86 fs. In bypass mode (with PLL disabled), the additive RMS jitter of each clock distributor was estimated to be about 16 fs at 1 GHz, resulting in approximately 26 fs RMS jitter at the ADC; i.e., after the second clock distributor. As the ADS54J40 has a typical internal aperture jitter of 120 fs RMS, the SNR and SFDR performance is mainly affected by the used A/D converters.

## 3. Digital Compensation of RF Impairments

Prior to digital predistortion, there were several transceiver impairments that needed to be compensated or at least taken into account, as listed below.

- Phase noise of local oscillators;
- Sampling clock jitter;
- Carrier frequency offset between TX and RX;
- Sampling frequency offset between TX and RX;
- DC offset at TX and RX;
- Gain and phase imbalance of TX and RX quadrature modulators dependent on frequency.

Phase noise and sampling clock jitter cannot be substantially compensated by any digital methods, except for the compensation of a constant rotation caused by phase noise [28], resulting in only marginal performance improvement. It is only possible to limit their effect by careful design. That was the reason why we used an ultra-low-phase-noise SAW oscillator at a fixed frequency as a clock source as described in Section 2.5.

**Figure 4:**
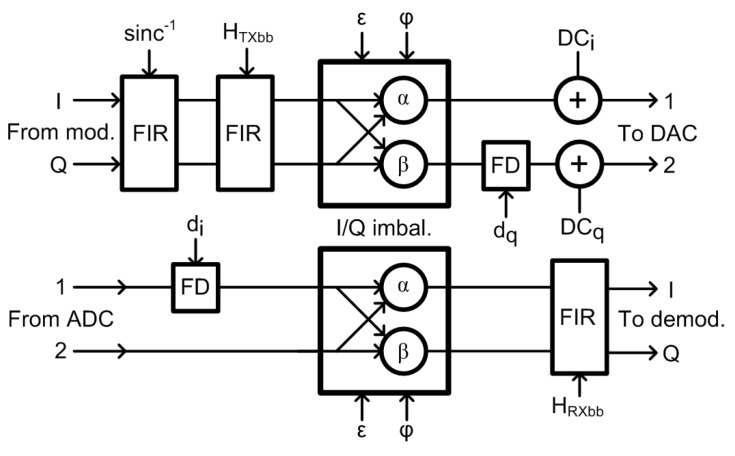
Digital baseband compensation blocks. (FIR: Finite Impulse Response. FD: Fractional Delay).

In general-purpose wireless transceivers, the carrier frequency offset and the sampling frequency offset between TX and RX represent serious issues [27]. In the designed test-bed, the RX part serves as the observation receiver [45,46] for DPD adaptation and there is neither carrier nor sampling frequency mismatch as the RF local oscillator and the clock source of A/D and D/A converters are shared for both TX and RX sides. We thus limit our description here mainly to DC offset and gain and phase imbalance compensation. Figure 4 shows the overall schematics of the implemented digital compensations for amplitude and phase mismatches and DC offset, all applied at digital baseband stages of TX and RX.

### 3.1. Gain and Phase Imbalance Compensation

Besides the compensation of imbalances induced by the quadrature modulators, the sin(x)/x roll-off of D/A converters have to be corrected. At the TX stage, this is done by an inverse *sinc* time domain Finite Impulse Response (FIR) filter through which the baseband samples are passed.

Due to the wide bandwidth of our test-bed, we assumed that the gain and phase mismatch of both transmitter and receiver are frequency-dependent. The effect of this mismatch on the transmitted signal is often expressed in the frequency domain as [47]

(1) $$X_{iq}\left(f\right)=\alpha \left(f\right)X\left(f\right)+\beta \left(f\right)X^{*}\left(-f\right)$$

with

(2) $$\alpha \left(f\right)=\frac{1+\left(1+\epsilon \left(f\right)\right)\exp^{j\phi (f)}}{2}$$

(3) $$\beta \left(f\right)=\frac{1-\left(1+\epsilon \left(f\right)\right)\exp^{j\phi (f)}}{2},$$

where X(f) and $X_{iq}\left(f\right)$ are components of the original, and the I/Q-impaired signal at frequencies *f*, ϵ(f) and ϕ(f) correspond to frequency-dependent gain and phase imbalances.

**Figure 5:**
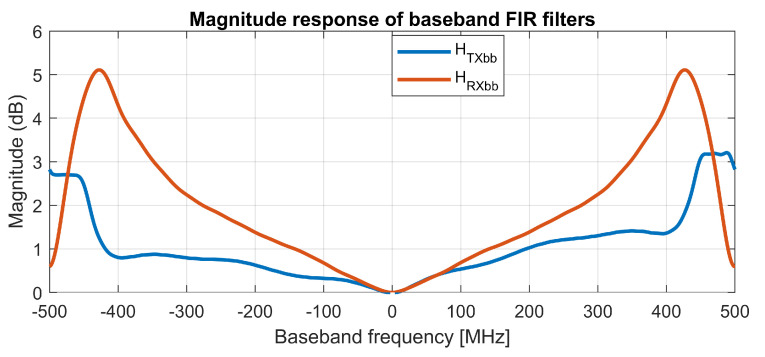
Frequency characteristics of baseband compensation filters $H_{TXbb}$ and $H_{RXbb}$.

**Figure 6:**
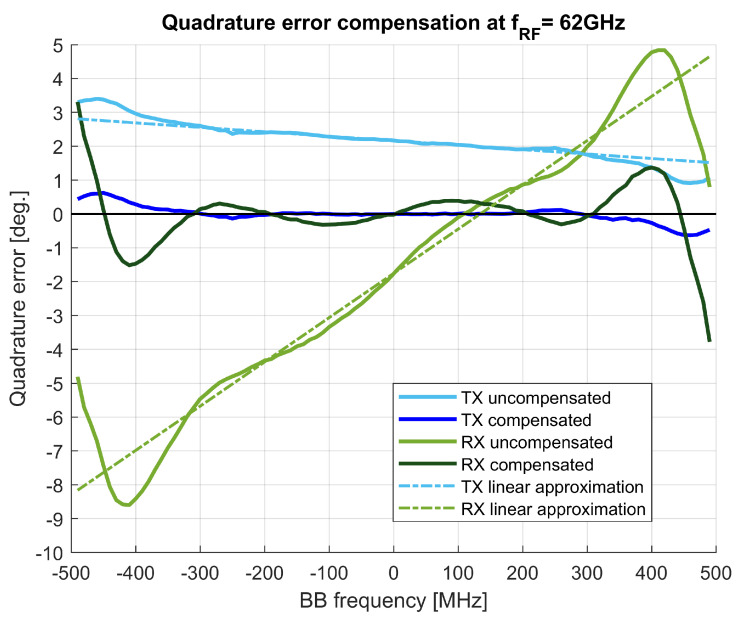
Quadrature error at TX and RX sides, with and without digital compensation.

In the described test-bed, the compensation of frequency-dependent gain and phase imbalances is done in three steps. First, the frequency dependent amplitude variations are corrected by an FIR filter $H_{TXbb}$ with the complex coefficients allowing for non-symmetrical frequency characteristic, with the measured magnitude response shown in Figure 5. For the second step, the frequency-independent imbalances of the quadrature I/Q modulator are corrected with the values obtained by the measurements summarized in Table 4. In the third step, a first-order frequency-dependent phase imbalance is corrected by a fractional delay $d_{q}$ introduced into the quadrature path of TX. The fractional delays $d_{q},d_{i}$ were calculated from slopes of linear approximations of the quadrature error. The measured phase imbalances (sometimes denoted as quadrature errors) before and after compensation are shown in Figure 6 for both the transmitter and the receiver.

At the receiver side, the compensation of imbalances is equivalent to that of the transmitter, but with an inverted order of operations. A fractional delay $d_{i}$ is inserted first, and then a frequency-independent I/Q compensation is applied; as a last step, the baseband amplitude response is corrected by an FIR filter with a frequency characteristic of $H_{RXbb}$.

To summarize, the employed compensation at the TX side reduced the frequency-dependent phase imbalance from the initial range of (+0.9,+3.4) degrees to approximately (−0.5,+0.5) degrees in the 900 MHz bandwidth. At the RX side, the initial phase imbalance in the range of (−7,+4) degrees was reduced to the range close to (−0.5,+0.5) degrees for a bandwidth of almost 700 MHz.

### 3.2. DC Offset Correction

The BGT60 transceiver supports internal DC offset settings [42] for the TX path. However, we found experimentally that setting TX DC compensation outside default (center) values degraded the baseband harmonic distortion characteristics. Therefore, the transmitter DC offset correction is applied to the baseband signal prior to sending the samples to DAC (see Figure 4), where it is denoted as ${\mathrm{DC}}_{i},{\mathrm{DC}}_{q}$.

On the contrary, DC offset at the receiver side is not corrected in the digital domain but is compensated by the current sources at the ADC driver amplifier inputs, meaning that DC offset is not amplified by the ADC drivers with high gain.

### 3.3. Overall Transfer Characteristics of the Transmitter-Observation Receiver Chain

**Figure 7:**
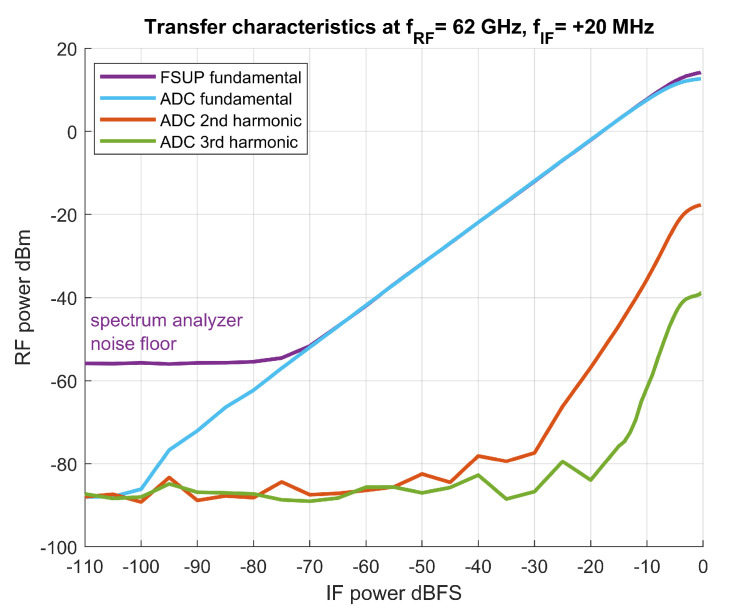
Overall measured transfer characteristics of the transmitter–observation receiver chain.

An example of the transfer characteristics of the whole transmitter–observation receiver chain at an RF frequency of 62 GHz is shown in Figure 7. The output power was also measured by an FSUP spectrum analyzer (a violet trace) and the observation receiver path gain was calculated and applied accordingly. At maximum power, the FSUP trace indicates that the transmitter is driven to approximately 2 dB compression. Additionally, ADC power and FSUP power traces are separated, indicating that the receiver is driven to compression (about 1.5 dB below the FSUP trace).

## 4. Digital Predistortion Experiments on Modulated Waveforms

### 4.1. Power Amplifiers

After the calibration of the test-bed, experiments investigating the digital predistortion of the power amplifiers were carried out. We considered two cases of PA configuration: in the first one, the internal PA of the BGT60 board driven above its 1 dB compression point was linearized; in the second case, the BGT60 internal PA was operated in a perfectly linear regime (only up to −17 dBm mean power at BGT60 PA output) driving the external PA QPW-50662330 from QuinStar. Basic parameters of these two amplification stages are summarized in Table 5.

### 4.2. Digital Predistortion

For low complexity, but yet satisfactory performance, we have considered a memory polynomial baseband digital predistorter [48]:

(4) $$y\left[n\right]=\sum_{p=1}^{P}\sum_{q=0}^{Q}{\hat{b}}_{p,q}x\left[n-q\right]{\left|x\left[n-q\right]\right|}^{p-1}$$

where x[n],y[n] are the predistorter input and output samples at time instant *n*, *P* describes the polynomial order, *Q* refers to the memory depth and ${\hat{b}}_{p,q}$ shows the PA model coefficients estimates. The predistorter coefficients were estimated by the standard least-squares method [48] with an indirect learning adaptation.

### 4.3. Time Synchronization

In addition to compensation of the impairments described in Section 3, a precise time alignment between the transmitted signal and the signal from the observation receiver needed to be ensured. This was not an issue in our previous narrowband 60 GHz setup [41] as triggering was provided by a dedicated CompuScope/CompuGen trigger signal. Despite the coherent clocks between the TX and RX paths in the current wideband test-bed, we needed to employ a precise time synchronization algorithm. As the transmitted signal is known in the envisaged DPD application, we employed a simple correlation between the transmitted and observed (received) signals for the initial time synchronization. Alternatively, some kind of preamble-based approach [49] could be used. For sub-sample time synchronization, we used the Fast Fourier Transform (FFT)-based approach [50].

### 4.4. Transmitted Signals

The performance of the test-bed with a predistorter was tested on three distinct signals: the M-ary Quadrature Amplitude Modulation (MQAM) with M = 16 states as a typical single-carrier signal, the Orthogonal Frequency Division Multiplexing (OFDM) as the most widespread multi-carrier signal, and the Filter Bank Multi Carrier (FBMC) as an example of a multi-carrier signal with decreased out-of-band radiation.

The root-raised cosine with a roll-off of 0.3 was used to shape the spectrum of the 16 QAM modulator. The number of samples per symbol was varied in selected discrete steps from 60 (resulting in approximately 20 MHz bandwidth) to 3 (more than 300 MHz bandwidth).

For FBMC, the parameters as per the PHYDYAS project [51] were used. For both OFDM and FBMC cases, the length of an inverse Fast Fourier Transform (FFT) in the modulator was set to a fixed value of 1024. To vary the bandwidth of these two waveforms, the number of data subcarriers $L_{\mathrm{data}}$ out of a total of *L* subcarriers was changed from 20 to 300 in steps that were selected to approach the same bandwidth as in the case of the single carrier 16 QAM signal. The remaining inputs of IFFT were zero-padded. The inner mapping on individual subcarriers of both multicarrier signals was 16 QAM.

### 4.5. Digital Predistortion Performance

The influence of DPD on the overall transmitter performance under modulated signals was evaluated by the EVM metric. We calculated the EVM value by averaging it over all $L_{\mathrm{data}}$ symbols transmitted on data subcarriers, and we expressed this in decibels:

(5) $${\bar{\mathrm{EVM}}}_{\mathrm{dB}}=20\cdot \log_{10}\left(\sqrt{\frac{\sum_{l\in L_{\mathrm{data}}}{\left({\hat{d}}_{l}-d_{l}\right)}^{2}}{\sum_{l\in L_{\mathrm{data}}}{\left(d_{l}\right)}^{2}}}\right),$$

where $L_{\mathrm{data}}$ is a set of all $L_{\mathrm{data}}$ data symbols and $d_{l},{\hat{d}}_{l}$ are ideal and received symbols, respectively. Note that for a fair comparison of linearized and unlinearized transmission, the PA without DPD has always been back-offed to the mean power equal to mean power of an amplifier with DPD.

**Figure 8:**
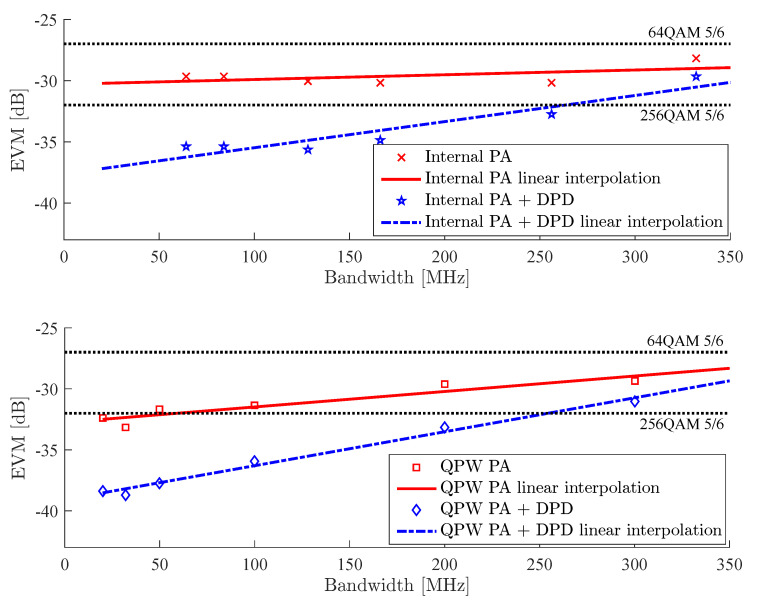
EVM with and without DPD for an OFDM waveform with an internal PA (top) and external QPW-50662330 PA (bottom). Dotted horizontal lines denote typical EVM limits for 64 Quadrature Amplitude Modulation (QAM) and 256 QAM with a code rate of 5/6.

The EVM results as a function of signal bandwidth for the two cases of power amplifiers operated at an RF frequency of 62 GHz and driven with an OFDM waveform are shown in Figure 8. For better orientation in the graphs, the discrete measured points denoted with markers have been linearly interpolated by dash-dotted (with DPD) and solid (without DPD) lines.

For reference, the black dotted horizontal lines represent the EVM limit requirements for two modulation and coding schemes (MCS, 64 QAM with a coding rate of 5/6 and 256 QAM with a coding rate if 5/6) imposed by a typical example of a contemporary OFDM-based WiFi system: IEEE 802.11ac. Note that, although this system is not defined for 60 GHz band operation, we chose it as it defines the EVM limits for 256 QAM, and for lower-order modulations, the limits are in good agreement with 60 GHz-based standards; see also Table 6.

We can observe that the maximal EVM improvement (around 7 dB) due to applying DPD was found for the narrowband signals, but even for the 300 MHz bandwidth multicarrier case, DPD provided a slight EVM improvement. Above this point, DPD had no positive effect on EVM. This well corresponds to a widely accepted rule of thumb of digital predistortion that states that the bandwidth of the signal in the observation receiver needs to be at least three times wider than the bandwidth of the original signal. For OFDM with 200 MHz bandwidth, the presented test-bed achieved comparable performance in terms of EVM as that in [16], which deliberately did not include the power amplifier at all and thus its trasmission power was much lower; see Table 1.

The EVM results for FBMC and single-carrier 16 QAM are summarized in Table 7 and Table 8, respectively. Again, the results at 62 GHz are presented for the internal PA as well as for the external QPW PA with and without DPD. The FBMC results share the same trend as the results for the OFDM waveform. The EVM requirement for 256 QAM was, due to DPD, fulfilled for both OFDM and FBMC up to a bandwidth of 250 MHz with BGT60’s internal PA. This also held for QPW PA with OFDM as an input, while for FBMC, the bandwidth allowing for 256 QAM with a coding rate of 5/6 was reduced to approximately 200 MHz. Note the performance of the EVM for both multicarrier signals was much less bandwidth-dependent, while for QAM, it degraded faster with growing bandwidth and for QPW PA, the requirements imposed by the most stringent MCS were fulfilled only for the 20 MHz case.

Keeping in mind a 1 dB compression point for the BGT60 internal PA of 12.2 dBm, with a Peak to Average Power Ratio (PAPR) of generated 16 QAM signals of approximately 7 dB and a PAPR of OFDM and FBMC signals of around 10 dB (depending on the used bandwidth and instantaneous random data realization), the mean output power was approximately 6.5 dBm in the case of 16 QAM and around 3.5 dBm in the case of both OFDM and FBMC.

Finally, we can conclude that, for a given transmitted power, the application of DPD provides us the potential of increasing the modulation order of OFDM/FBMC subcarriers from the recommended 64 QAM [42] to 256 QAM for a bandwidth of up to 250 MHz. With its transmission/reception bandwidth of 900 MHz without DPD, the test-bed is ready to be used for future evaluations of 5G NR Release 17 waveforms with a mandatory subcarrier spacing of 120 kHz and a maximum number of 275 resource blocks; i.e., 400 MHz of bandwidth. According to the recent report from ongoing 5G NR standardization tests [5], some companies have observed that the support of channel bandwidths such as 200 or 400 MHz may enable the efficient usage of the available spectrum. The presented test-bed could thus also be used in such evaluations, focusing on research into PA efficiency enhancement techniques and DPD. Another envisaged application is the research into the linearization of 60 GHz active antennas, similarly to the recent experiment [35] in the 28 GHz band, with the challenging aspect of multiple PA estimation based on the single observation receiver. Note that although we designed the D/A converter board to allow it to be extended to two channels, the BGT60 modules with integrated VCO [42] prohibit its direct use in a two-channel multiple input–multiple output (MIMO) scenario. Nevertheless, the described test-bed can serve as a two-channel transceiver, e.g., for research into the hot topic of user interference due to beam misalignment [53].

**Figure 9:**
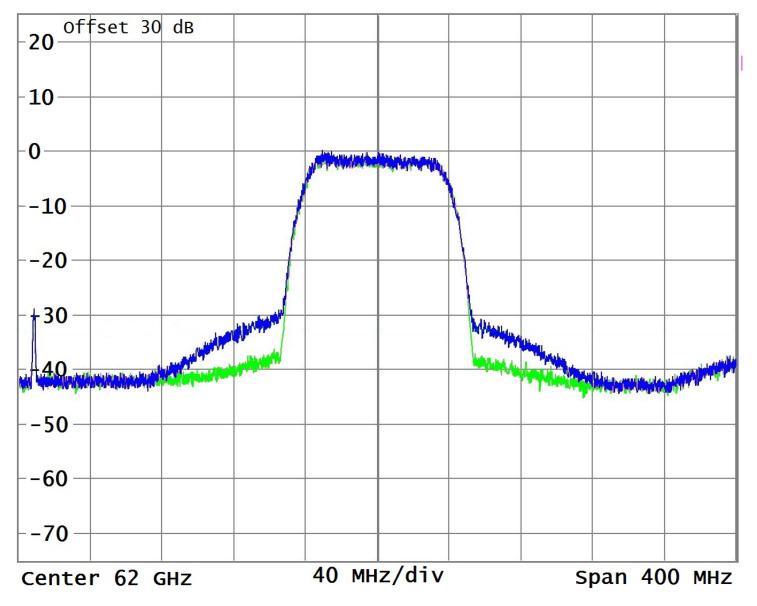
Example of the measured spectrum of a 16 QAM signal at a QPW power amplifier output with (green) and without (blue) DPD.

Besides the reduction of in-band distortions that we have demonstrated on EVM curves, the out-of-band emissions caused by the nonlinearity were reduced by DPD. Although the out-of-band emissions are not as important in the 60 GHz band due to the spatial separation of users in mm-waves, we investigated the effect of predistortion to a transmitted spectrum. An example of a spectrum measured at the QPW PA output with and without predistortion is shown in Figure 9 for a 16 QAM signal with a 100 MHz bandwidth. Note that due to the limited sensitivity of the spectrum analyzer with an external harmonic mixer, we are not able to present the spectrum results for wider bandwidths.

## 5. Conclusions

In millimeter waves, the experimental evaluation of RF front-end impairment compensation techniques is currently predominantly performed using expensive, universal instrumentation or by computer simulations. In this paper, we have presented a detailed description of our fully MATLAB-controlled experimental wideband test-bed for the 60 GHz band. The described test-bed was carefully designed for low phase noise and sampling clock jitter and is equipped with a coherent RF observation path to facilitate the estimation of RF front-end impairments. After compensation for estimated gain and phase variations and DC offset calibration, the test-bed can be used to research digital predistortion algorithms for millimeter-wave power amplifier linearization.

The functionality of the developed test-bed was evaluated on three typical communication signals: a single carrier MQAM and two cases of multi-carrier signals—OFDM and FBMC. Regarding Error Vector Magnitude performance, we have demonstrated the advantage of the linearization of the internal as well as of the external medium-power 60 GHz amplifier by means of digital predistortion in a wide range of signal bandwidths. For both amplifiers in the test, we have shown that digital linearization allows the use of higher-order modulations and thus the overall data throughput can be substantially increased.

## Figures and Tables

**Table 1:** 60 GHz test-bed comparisons. TX: transmitter; RF: radio frequency; DPD: digital predistortion; OFDM: Orthogonal Frequency Division Multiplex; EVM: Error Vector Magnitude.

Reference	Max. TX	Max. RF	Real-Time	DPD	OFDM Sig.	EVM
	Bandwidth	CW Power			Bandwidth	
	(MHz)	(dBm)			(MHz)	(dB)
Quadri [38]	25	12	Yes	No	25	−20
Duarte [16]	1250	~1	Yes	No	122	−33
This work	900	24	No	Yes	200	−32.2

**Table 2:** Phase noise of the BGT60 transmitter at 60 GHz.

Offset	Datasheet Value	Measured Value
(Hz)	(dBc/Hz)	(dBc/Hz)
10 k	N/A	−48
100 k	−84	−80
1 M	−104	−102
10 M	−124	−120

**Table 3:** Phase noise of clock sources at 1 GHz. (PLL: Phase Locked Loop).

Offset	PLL Solution	Fixed. Freq. Solution
(Hz)	(dBc/Hz)	(dBc/Hz)
1 k	−112	−112
10 k	−123	−142
100 k	−124	−155
1 M	−138	−167
10 M	−151	−168
**RMS Jitter (1 kHz–20 MHz)**		
PLL solution		86 fs
SAW osc. itself		13.5 fs
SAW osc.+distributors		26.3 fs

**Table 4:** Measured frequency-independent I/Q compensation parameters at 62 GHz.

	Amplitude Imbalance	Quadrature Imbalance	Fractional Delay
	ε [dB]	φ [deg.]	$d_{i}\left(\mathrm{RX}\right),d_{q}\left(\mathrm{TX}\right)$ [ps]
TX path	−0.44	+2.14	7.62
RX path	0.17	−1.75	72.0

**Table 5:** Parameters of power amplifiers at 62 GHz.

	Frequency Range	$P_{1\mathrm{dB}}$ at 62 GHz	Gain at 62 GHz
	(GHz)	(dBm)	(dB)
BGT60 internal PA	57–64	12.2	32
QuinStar QPW-50662330 PA	50–66	24	35

**Table 6:** EVM limits for a typical exemplary OFDM system: 802.11ac. [52]

Modulation Scheme	Code Rate	EVM Limit (802.11ac)
BPSK	1/2	−5 dB
QPSK	1/2	−10 dB
QPSK	3/4	−13 dB
16 QAM	1/2	−16 dB
16 QAM	3/4	−19 dB
64 QAM	2/3	−22 dB
64 QAM	3/4	−25 dB
64 QAM	5/6	−27 dB
256 QAM	3/4	−30 dB
256 QAM	5/6	−32 dB

**Table 7:** EVM with and without digital predistortion for FBMC as a function of the number of data subcarriers $L_{\mathrm{data}}$. The symbol ${\left(\right)}^{1}$ denotes the EVM fulfilling requirement for 256 QAM and a code rate of 5/6.

$L_{\mathrm{data}}$	Bandwidth	EVM	EVM	EVM	EVM
		(Internal PA)	(Internal PA+DPD)	(QPW PA)	(QPW PA+DPD)
	(MHz)	(dB)	(dB)	(dB)	(dB)
50	50.7	-	-	−28.4	−34.9
64	64.4	−29.6	−32.4	-	-
100	99.6	-	-	−29.0	−34.4
128	126.9	−29.1	−32.4	-	-
200	197.3	-	-	−28.6	$-32.0^{1}$
250	246.1	−28.1	$-32.0^{1}$	-	-
330	324.2	−27.7	−31.7	−28.2	−30.7

**Table 8:** EVM with and without digital predistortion for 16 QAM as a function of samples per symbol (SpS).

SpS	Bandwidth	EVM	EVM	EVM	EVM
		(Internal PA)	(Internal PA+DPD)	(QPW PA)	(QPW PA+DPD)
[-]	(MHz)	(dB)	(dB)	(dB)	(dB)
64	20	−31.1	−36.5	−27.1	−32.0
12	100	−27.6	−36.1	−26.4	−31.9
8	150	−25.5	−34.4	−26.0	−31.4
6	200	−23.6	−32.4	−25.0	−30.7
4	300	−15.5	−16.9	−11.1	−11.1

## Data Availability

An example data set containing the modulated waveforms and acquired QPW power amplifier output data after time synchronization have been put into a public repository https://github.com/romanmarsalekBUT/60GHz_PA_data. We hope they will find usage in experiments with PA modeling.

## References

[1] Weiler R.J., Peter M., Keusgen W., Calvanese-Strinati E., De Domenico A., Filippini I., Capone A., Siaud I., Ulmer-Moll A., Maltsev A. Enabling 5G backhaul and access with millimeter-waves. Proceedings of the 2014 European Conference on Networks and Communications (EuCNC).

[2] Azpilicueta L., Lopez-Iturri P., Zuñiga-Mejia J., Celaya-Echarri M., Rodríguez-Corbo F.A., Vargas-Rosales C., Aguirre E., Michelson D.G., Falcone F. (2020). Fifth-Generation (5G) mm Wave Spatial Channel Characterization for Urban Environments’ System Analysis. Sensors.

[3] Blazek T., Zöchmann E., Mecklenbräuker C. (2018). Millimeter Wave Vehicular Channel Emulation: A Framework for Balancing Complexity and Accuracy. Sensors.

[4] Shimodaira H., Kim J., Sadri A.S. (2016). Enhanced Next Generation Millimeter-Wave Multicarrier System with Generalized Frequency Division Multiplexing. Int. J. Antennas Propag..

[5] 3GPP TR 38.808 V0.2.0 (2020) 3rd Generation Partnership Project; Technical Specification Group Radio Access Network; Study on supporting NR from 52.6 GHz to 71 GHz (Release 17). https://portal.3gpp.org/desktopmodules/Specifications/SpecificationDetails.aspx?specificationId=3735.

[6] El-Absi M., Zheng F., Abuelhaija A., Al-haj Abbas A., Solbach K., Kaiser T. (2020). Indoor Large-Scale MIMO-Based RSSI Localization with Low-Complexity RFID Infrastructure. Sensors.

[7] Bellanger M. Filter banks and OFDM-OQAM for high throughput wireless LAN. Proceedings of the 2008 3rd International Symposium on Communications, Control and Signal Processing.

[8] Michailow N., Matthé M., Gaspar I.S., Caldevilla A.N., Mendes L.L., Festag A., Fettweis G. (2014). Generalized Frequency Division Multiplexing for 5th Generation Cellular Networks. IEEE Trans. Commun..

[9] Jayati A.E., Suryani T. (2019). Nonlinear Distortion Cancellation using Predistorter in MIMO-GFDM Systems. Electronics.

[10] Zhang X., Jia M., Chen L., Ma J., Qiu J. Filtered-OFDM—Enabler for Flexible Waveform in the 5th Generation Cellular Networks. Proceedings of the 2015 IEEE Global Communications Conference (GLOBECOM).

[11] Choi H. (2019). Class-C Linearized Amplifier for Portable Ultrasound Instruments. Sensors.

[12] Gotthans T., Maršálek R., Blumenstein J., Baudoin G. Experimental evaluation of digital predistortion with FBMC and OFDM signals. Proceedings of the 2015 IEEE 16th Annual Wireless and Microwave Technology Conference (WAMICON).

[13] Zayani R., Shaiek H., Alexandre C., Kielys A., Cheng X., Fu X., Roviras D. A Testbed for Experimental Performance Evaluation of Multicarrier Waveforms in Presence of RF PA. Proceedings of the 2018 15th International Symposium on Wireless Communication Systems (ISWCS).

[14] Zayani R., Shaiek H., Cheng X., Fu X., Alexandre C., Roviras D. (2018). Experimental Testbed of Post-OFDM Waveforms Toward Future Wireless Networks. IEEE Access.

[15] Nissel R., Zöchmann E., Lerch M., Caban S., Rupp M. Low-latency MISO FBMC-OQAM: It works for millimeter waves! In Proceedings of the 2017 IEEE MTT-S International Microwave Symposium (IMS), Honololu, HI, USA, 4–9 June 2017.

[16] Duarte L., Gomes R., Ribeiro C., Caldeirinha R.F.S. (2019). A Software-Defined Radio for Future Wireless Communication Systems at 60 GHz. Electronics.

[17] Gomes R., Reis J., Al-Daher Z., Hammoudeh A., Caldeirinha R.F.S. (2018). 5G: performance and evaluation of FS-FBMC against OFDM for high data rate applications at 60 GHz. IET Signal Process..

[18] Choi H., Woo P.C., Yeom J.Y., Yoon C. (2017). Power MOSFET Linearizer of a High-Voltage Power Amplifier for High-Frequency Pulse-Echo Instrumentation. Sensors.

[19] Gharaibeh K.M., Gard K.G., Steer M.B. Accurate estimation of digital communication system metrics—SNR, EVM and /spl rho/ in a nonlinear amplifier environment. Proceedings of the 64th ARFTG Microwave Measurements Conference, Fall 2004.

[20] Suryasarman P.M., Springer A. (2015). A Comparative Analysis of Adaptive Digital Predistortion Algorithms for Multiple Antenna Transmitters. IEEE Trans. Circuits Syst. I Regul. Pap..

[21] Aquino G., Guimarães D., Mendes L., Pimenta T. (2017). Combined Pre-Distortion and Censoring for Bandwidth-Efficient and Energy-Efficient Fusion of Spectrum Sensing Information. Sensors.

[22] Vasjanov A., Barzdenas V. (2018). A Review of Advanced CMOS RF Power Amplifier Architecture Trends for Low Power 5G Wireless Networks. Electronics.

[23] Tsai J., Huang T. (2007). 35–65-GHz CMOS Broadband Modulator and Demodulator With Sub-Harmonic Pumping for MMW Wireless Gigabit Applications. IEEE Trans. Microw. Theory Tech..

[24] Krone S., Fettweis G. Capacity Analysis for OFDM Systems with Transceiver I/Q Imbalance. Proceedings of the IEEE GLOBECOM 2008—2008 IEEE Global Telecommunications Conference.

[25] Marsalek R., Blumenstein J., Pospisil M., Rupp M. Measured Capacity of mm-Wave Radio Link Under IQ Imbalance. Proceedings of the 2018 IEEE 29th Annual International Symposium on Personal, Indoor and Mobile Radio Communications (PIMRC).

[26] Choi H., Yoon C., Yeom J.Y. (2017). A Wideband High-Voltage Power Amplifier Post-Linearizer for Medical Ultrasound Transducers. Appl. Sci..

[27] Bogdan R., Balsara P. (2006). All-Digital Frequency Synthesizer in Deep-Submicron CMOS.

[28] Garcia Armada A. (2001). Understanding the effects of phase noise in orthogonal frequency division multiplexing (OFDM). IEEE Trans. Broadcast..

[29] Mallet C., Duvanaud C., Carré L., Bachir S. Analog predistortion for high power amplifier over the Ku-band (13,75–14,5 GHz). Proceedings of the 2017 47th European Microwave Conference (EuMC).

[30] Li J., Salmon N.A., Gumbmann F. (2020). Millimetre-wave beam steering with analog-resolution and minimised distortion based on liquid crystals tunable delay lines with enhanced signal-to-noise ratios. Millimetre Wave and Terahertz Sensors and Technology XIII.

[31] Razavi B. (1996). Challenges in portable RF transceiver design. IEEE Circuits Devices Mag..

[32] Chung A., Ben Rejeb M., Beltagy Y., Darwish A.M., Hung H.A., Boumaiza S. (2018). IQ Imbalance Compensation and Digital Predistortion for Millimeter-Wave Transmitters Using Reduced Sampling Rate Observations. IEEE Trans. Microw. Theory Tech..

[33] Beltagy Y., Chung A., Mitran P., Boumaiza S. On the calibration of the feedback receiver using reduced sampling rate and its application to digital predistortion of 5G power amplifiers. Proceedings of the 2017 IEEE MTT-S International Microwave Symposium (IMS).

[34] Kovacic S. (2017). Wideband Millimeter Wave Test Bed for 60 GHz Power Amplifier Digital Predistortion. Microw. J.

[35] Campo P.P., Brihuega A., Anttila L., Turunen M., Korpi D., Allén M., Valkama M. (2021). Gradient-Adaptive Spline-Interpolated LUT Methods for Low-Complexity Digital Predistortion. IEEE Trans. Circuits Syst. I Regul. Pap..

[36] Miyanaga K., Kobayashi M., Saito N., Shirakata N., Takinami K. (2016). A Wideband Asymmetric Digital Predistortion Architecture for 60 GHz Short Range Wireless Transmitters. IEICE Trans. Electron..

[37] Vychodil J., Pospisil M., Prokes A., Blumenstein J. (2019). Millimetre wave band time domain channel sounder. IET Commun..

[38] Quadri A., Zeng H., Hou Y.T. A Real-Time mmWave Communication Testbed with Phase Noise Cancellation. Proceedings of the IEEE INFOCOM 2019—IEEE Conference on Computer Communications Workshops (INFOCOM WKSHPS).

[39] Maršálek R., Pospíšil M., Gotthans T., Urbanec T. Digital Calibration of 60 GHz Setup for Use in Power Amplifier Predistortion. Proceedings of the 2019 IEEE 20th International Workshop on Signal Processing Advances in Wireless Communications (SPAWC).

[40] Kral J., Gotthans T., Marsalek R., Harvanek M., Rupp M. (2020). On Feedback Sample Selection Methods Allowing Lightweight Digital Predistorter Adaptation. IEEE Trans. Circuits Syst. I: Regul. Pap..

[41] Maršálek R., Pospíšil M., Gotthans T., Urbanec T. 60 GHz Setup for RF Components Impairments Compensation. Proceedings of the 2018 IEEE MTT-S International Microwave Workshop Series on 5G Hardware and System Technologies (IMWS-5G).

[42] Infineon Technologies AG Single-Chip SiGe Transceiver Chipset for V-band Backhaul Applications from 57 to 64 GHz, Application note AN 376, Revision: Rev. 1.0. https://www.infineon.com/dgdl/Infineon--AN-v01_00-NA.pdf?fileId=5546d4624ad04ef9014aed1c06120a5e.

[43] Urbanec T., Maršálek R. Single plane V-band directional coupler for predistortion compensation. Proceedings of the 2018 28th International Conference Radioelektronika (RADIOELEKTRONIKA).

[44] Kobayashi H., Morimura M., Kobayashi K., Onaya Y. Aperture jitter effects in wideband sampling systems. Proceedings of the 16th IEEE Instrumentation and Measurement Technology Conference (Cat. No.99CH36309).

[45] Kral J., Gothans T., Marsalek R., Harvanek M. Digital Predistorter with Real-Valued Feedback Employing Forward Model Estimation. Proceedings of the 2018 25th International Conference on Telecommunications (ICT).

[46] Song Z., Liu X., Zhao X., Liu Q., Jin Z., Chi B. (2017). A Low-Power NB-IoT Transceiver with Digital-Polar Transmitter in 180-nm CMOS. IEEE Trans. Circuits Syst. I Regul. Pap..

[47] Kim M., Maruichi Y., Takada J. (2013). Parametric Method of Frequency-Dependent I/Q Imbalance Compensation for Wideband Quadrature Modulator. IEEE Trans. Microw. Theory Tech..

[48] Kim J., Konstantinou K. (2001). Digital predistortion of wideband signals based on power amplifier model with memory. Electron. Lett..

[49] Schmidl T.M., Cox D.C. (1997). Robust frequency and timing synchronization for OFDM. IEEE Trans. Commun..

[50] Kral J., Gotthans T., Harvanek M. Analytical method of fractional sample period synchronisation for digital predistortion systems. Proceedings of the 2017 27th International Conference Radioelektronika (RADIOELEKTRONIKA).

[51] M.Bellanger FBMC Physical Layer: A Primer. http://www.ict-phydyas.org/teamspace/internal-folder/FBMC-Primer_06-2010.pdf.

[52] National Instruments Introduction to WLAN Testing. http://download.ni.com/evaluation/rf/Introduction_to_WLAN_Testing.pdf.

[53] Grannemann L., Ichkov A., Mähönen P., Simić L. (2020). Urban Outdoor Measurement Study of Phased Antenna Array Impact on Millimeter-Wave Link Opportunities and Beam Misalignment. IEEE Trans. Wirel. Commun..

